# Improving therapy in metastatic uveal melanoma by understanding prior failures

**DOI:** 10.18632/oncoscience.510

**Published:** 2020-06-01

**Authors:** Daniel J. Olson, Jason J. Luke

**Affiliations:** ^1^University of Chicago Comprehensive Cancer Center, Chicago, IL, USA; ^2^UPMC Hillman Cancer Center, UPMC, Pittsburgh, PA, USA

**Keywords:** uveal melanoma, cabozantinib, targeted therapy, immunotherapy

Metastatic uveal melanoma (UM) has historically been a particularly difficult sub-set of disease to treat in the metastatic setting. Multiple early phase clinical trials of chemo-, targeted and immunotherapies have consistently failed to demonstrate convincing efficacy signals, and overall survival outcomes remain poor [1]. Despite negative results, these studies do set benchmark clinical outcome standards [2] and answer important scientific questions within the field. This allows researchers to shift efforts towards potentially more promising strategies and therapies that may ultimately benefit patients. One issue that has potentially limited the field to date has been a reliance upon primary disease to design therapeutic approaches in the metastatic setting. Recently The Cancer Genome Atlas [3] has described the genomics of primary UM, however studies of metastatic disease are only starting to emerge [4-6]. While analyses of primary UM have been insightful in the prognostication of primary tumor features associated with higher risks of metastases and poor clinical outcomes [7], these efforts may not have been optimal toward elucidating the next generation of therapeutic targets in metastatic UM .

These concepts around drug development in UM were, in part, illustrated by the recently-published, “Randomized Phase II Trial and Tumor Mutational Spectrum Analysis from Cabozantinib versus Chemotherapy in Metastatic Uveal Melanoma (Alliance A091201)” [8]. The overexpression of the tyrosine kinase MET from primary UM biospecimens, which associated with a significantly higher risk of death from metastatic UM and could be blocked in UM cell lines [9], served as a rationale for the study. Within the clinical trial, MET was targeted by cabozantinib, a small molecule inhibitor of multiple tyrosine kinases, including VEGFR2 and MET. The trial randomized patients with metastatic UM 2:1 to cabozantinib versus standard of care chemotherapy with temozolomide or dacarbazine. The primary endpoint of the study was progression-free survival at four months (PFS4), where a null hypothesis PFS4 of 15% was tested against an alternate PFS4 of 40%. Secondary endpoints included overall survival (OS), RECIST response rate, and safety. Ultimately, no differences in PFS4, PFS, or OS were observed and toxicity was found to be higher in the cabozantinib arm relative to chemotherapy. An exploratory analysis using whole exome sequencing of metastatic tumor specimens from trial patients was also completed. This revealed both well described (e.g. GNAQ, GNA11, BAP1, SF3B1), and lesser known mutations, and also revealed an average total tumor mutational burden (TMB) of 46 mutations/megabase (mut/Mb) across metastatic UM tumors. This was substantially lower than the what is commonly described for cutaneous melanoma (e.g. > 400 mut/Mb), highlighting the unique mutational profile of UM.

The molecular profile of UM, largely defined through primary UM biospecimens, also has informed previous clinical trials targeting the RAS-ERK pathway, which remains constitutively activated in patients with GNAQ/GNA11 mutations. Within the RAS-ERK pathway, MEK inhibition demonstrated early activity with the introduction of selumetinib, an oral selective MEK1/2 inhibitor. In an open-label phase II study comparing selumetinib to temozolomide or dacarbazine, median PFS was improved in the selumetinib arm [10]. This prompted the larger phase III SUMIT study where selumetinib was combined with chemotherapy and compared to chemotherapy alone; however, selumetinib failed to meet its primary PFS endpoint: no significant difference was observed for selumetinib as compared to chemotherapy alone (PFS 2.8 vs. 1.8 months, p = 0.32) with minimal associated response rates (3.1% vs. 0%) between selumetinib and chemotherapy [11]. Additionally, multiple phase I and phase II studies have failed to show substantial clinical benefit when targeting other molecular targets upstream, such as Protein Kinase C6, and downstream within the mitogen-activated protein kinase (MAPK) pathway [12-14]. Other molecular targets in UM have also been tested in clinical trials, but have similarly yielded very limited clinical activity; this includes VEGF (bevacizumab), c-KIT (imatinib, sunitinib) and epidermal growth factor inhibitors (gefitinib) [15-17].

The introduction of immunotherapy offers a different potential strategy for the treatment metastatic UM. Unlike cutaneous melanoma, immunotherapy in UM will likely rely on distinct modalities as initial outcomes with immune checkpoint inhibitors (ICIs) have been disappointing. To date, multiple case series and early phase trials have evaluated PD1 and CTLA4 antibodies for metastatic UM – alone or in combination – and have consistently demonstrated limited clinical benefit and low response rates [15-17]. For example, in a recent phase II trial, treatment with combined PD1 and CTLA4 antibodies generated a 17% response rate – one of the highest response rates reported across multiple early phase studies - while in cutaneous melanoma, response rates of 58% have been with the same regimen [18, 19]. Some evidence suggests that checkpoint blockade approaches might be useful in the subset of patients with low volume or non-hepatic involved UM, though such patients are relatively rare [20]. Despite these outcomes with ICIs, some encouraging data is emerging that UM can be successfully targeted with alternate immunotherapy modalities. A recent phase II trial in metastatic UM where autologous tumor-infiltrating lymphocytes (TIL) and high-dose interleuikin-2 were infused after lympho-depleting chemotherapy demonstrated a 35% (7/20) response rate [21]. Additionally, tebentafusp, a first-in-class immune-mobilizing monoclonal T cell receptor against cancer (ImmTAC), which is made up of a human leukocyte antigen-A*02:01 restricted soluble T cell receptor fused to an anti-CD3 single-chain variable fragment, has demonstrated early signs of activity in UM. Tabentafusp recruits CD3+ T cells to target cells that express the melanoma-associated antigen gp100, thus re-directing T cells towards melanoma cells. A phase 1 study of tebentafusp in UM showed one-year overall survival of 74%, which is numerically superior to the one-year overall survival published for historical treatment modalities in UM that have not surpassed 55% [22]. While these efficacy signals are still in early phases of testing, they do suggest that UM may ultimately be amenable to targeting by immunotherapy. As the understanding of the likely unique immunobiology of UM improves, the hope remains that these promising therapies translate into improvements in clinical outcomes for patients with UM.

Finally, another note of optimism for the treatment of UM is the integration of multi-omic technologies in the development of potential drug targets. Such technologies generate large amounts of genomic, epigenomic and proteomic data from UM biospecimens, and along with bioinformatic pipelines, can greatly improve collective knowledge around UM oncogenesis [3, 5, 23]. This improved understanding of the oncogenesis of UM can potentially identify molecular weaknesses and potential drug targets possibly including the recently identified Hippo/YAP pathway as potentially a high priority target in UM [24]. This approach also applies to immunotherapy approaches in UM where an improved understanding of the unique immunobiology of UM may offer more potential drug targets and treatment strategies to improve clinical outcomes for patients with metastatic UM.
